# A comparative analysis of the relationship between flood experience and private flood mitigation behaviour in the regions of England

**DOI:** 10.1111/jfr3.12700

**Published:** 2021-02-06

**Authors:** Marlies H. Barendrecht, Simon McCarthy, Alberto Viglione

**Affiliations:** ^1^ Centre for Water Resource Systems Vienna University of Technology Vienna Austria; ^2^ Flood Hazard Research Centre Middlesex University London UK; ^3^ Department of Environment, Land and Infrastructure Engineering Politecnico di Torino Torino Italy

**Keywords:** flood risk management, hierarchical beta regression, preparedness, property level measures

## Abstract

There has been a move towards a more integrated approach to flood risk management, which includes a stronger focus on property level measures. However, in England the uptake of these measures remains low. Flood experience has been found to influence preparedness (i.e., the uptake of measures), but even experience does not always result in an increase in preparedness. We investigate the variations in the relationship between experience and preparedness for the regions of England as defined by the Environment Agency. Analysis of survey data collected by the Environment Agency among the at risk population between 1997 and 2004 was undertaken to determine the differences between the seven regions. We find that in the South West, Southern and Anglian regions increases in preparedness with increasing experience are higher compared to other regions. In the Thames, Midlands and North West regions the preparedness increases less with increasing experience. We explore the influence of other factors influencing flood mitigation behaviour that have been previously found in the literature and find that the differences between regions are correlated with the severity of experienced flooding and whether English is the first language of the respondents.

## 1 INTRODUCTION

### 1.1 Flood experience and private property level flood preparedness

In recent years, there has been a shift in flood risk management from a focus on structural protection to a more integrated approach to reducing risk (Bubeck et al., 2017; European Union, 2007; Kuhlicke et al., 2020). This is accompanied by a shift of responsibilities from the government to the people (Johnson & Priest, 2008; Soane et al., 2010). There is an increasing focus on private flood protection whereby households and businesses are expected to take measures to prepare for a flood event and thus reduce losses in case flooding occurs (Bubeck, Botzen, Kreibich, & Aerts, 2013). Measures include, for example, moving valuables upstairs or the use of mobile flood barriers, but also preparing a plan that lists what to do should flooding occur (Kreibich, Bubeck, Van Vliet, & De Moel, 2015). Even though these kind of measures are considered a viable method to reduce risk by flood risk managers, the uptake of measures by residents is still very low (Everett & Lamond, 2013; Joseph, Proverbs, & Lamond, 2015; Kreibich, Seifert, Merz, & Thieken, 2010; Owusu, Wright, & Arthur, 2015). In order to better inform flood risk management and policies, it is necessary to improve our understanding of behaviour at the property level. Investigating the factors that can influence the uptake of private protection can help design measures and policies to influence this behaviour.

Several studies have found that there is a relationship between flood experience and the uptake of private measures (e.g., Bradford et al., 2012; Bubeck, Botzen, Kreibich, et al., 2012; Kreibich et al., 2010; Kreibich & Thieken, 2009; Miceli, Sotgiu, & Settanni, 2008; Osberghaus, 2015; Owusu et al., 2015; Poussin, Botzen, & Aerts, 2014). When people have experienced flooding before, they are more likely to take measures to be prepared in case of a future event. However, the relationship between preparedness and experience is influenced by other factors as well. Not every resident that has experienced flooding, also takes measures as a consequence of this experience. In the rest of this article, we use the term preparedness or private measures, to indicate measures that are taken at a property level to mitigate flood risk. These measures include physical measures, like sandbags or installing airbricks but also measures like registering for flood warnings or preparing a plan listing what to do should flooding occur.

Thieken, Kreibich, Müller, and Merz (2007) find that not only experience itself influences the uptake of private measures, but also the impact of this experience is important. The presence of structural protection or the construction of structural defences immediately following an event may provide a false sense of security that reduces the uptake of private measures (Hanger et al., 2018; Scolobig, De Marchi, & Borga, 2012) although Poussin et al. (2014) find that the uptake of measures is positively influenced by the presence of structural protection. Scolobig et al. (2012) and Duží, Vikhrov, Kelman, Stojanov, and Juřička (2017) find that financial costs or a lack of knowledge about which measures to take may hinder the uptake of private measures. Therefore, provision of information or financial incentives (either through governmental support or from insurance) can increase the uptake of measures (Hanger et al., 2018; Poussin et al., 2014; Thieken et al., 2007). A strong social network (Poussin et al., 2014) and household size (Thieken et al., 2007) may also influence the preparedness positively, possibly because people that feel supported may have an increased belief in their ability to mitigate the risk. Other factors that have been found to influence the uptake of measures are home ownership (Thieken et al., 2007), number of men and children in a household (Duží et al., 2017), elevation of the property (Duží et al., 2017), closeness to a water course (Miceli et al., 2008) and respondent's age (Miceli et al., 2008).

To advance our knowledge about human‐flood systems it is valuable to compare different case studies. This may lead to further understanding of the differences in behaviour of residents and why some do increase their preparedness, while others do not. However, comparing the results across case studies is difficult because the studies and surveys may not have been set up in the same way. Also, the flood risk management and governance system that these case studies are part of are not the same. To improve our understanding of the differences in the relationship between flood experience and the uptake of measures, it would be valuable to contrast and compare different cases with each other that are part of the same flood risk management system and that have been surveyed in a consistent manner. This is the focus of this study. We compare different cases in England to determine the differences in the relationship between experience and preparedness.

### 1.2 Property level flood loss mitigation in England

**FIGURE 1:**
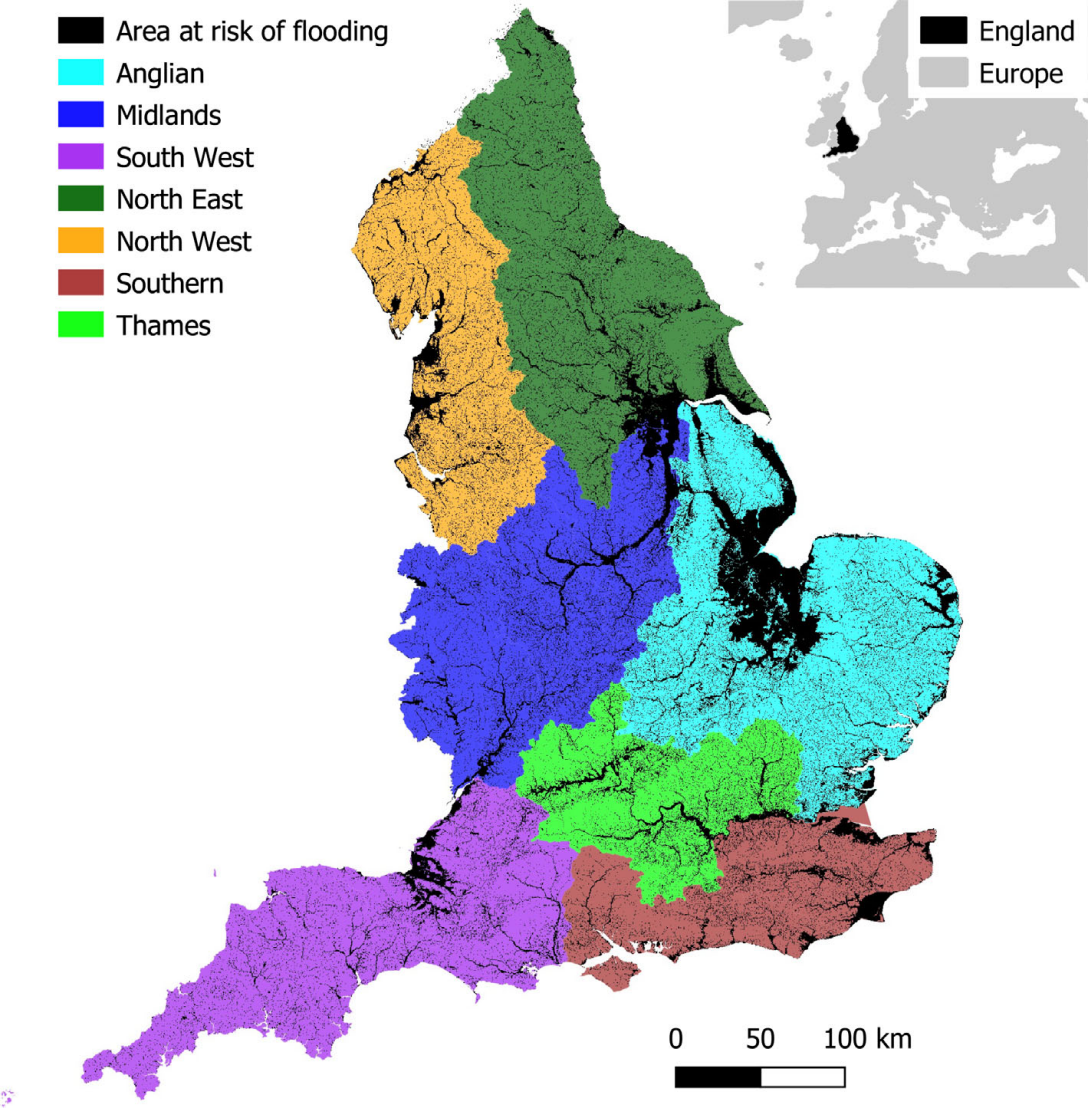
The seven regions of England as previously used by the Environment Agency, with in black the area that is at risk of coastal or riverine flooding. Region outlines and Area at risk of flooding from Environment Agency (2006, 2016). © EuroGeographics for the administrative boundaries of Europe

In England, one in twelve properties are estimated to be at risk of flooding from fluvial and tidal sources (Fielding, 2017). This number may increase up to one in six when other sources of flooding like surface water flooding are taken into account (Fielding, 2017). The country is frequently hit by floods and the estimated annual flood damage is £1 billion (Harries & Penning‐Rowsell, 2011). England has an integrated approach to flood risk management. Instead of only relying on structural protection to reduce the risk of flood inundation, non‐structural measures are included in flood risk management plans (Bubeck et al., 2017; Harries, McEwen, & Wragg, 2018). The main responsible agency for the management of flood risk in England is the Environment Agency (Bubeck et al., 2017). In the last decades the Environment Agency has attempted to raise awareness of flood risk among the at risk population in England and to increase their knowledge of what to do in case flooding occurs. The Agency has monitored, through surveys, the awareness and understanding of flood risk, as well as the uptake of measures to mitigate flood risk, in the different regions of England (Figure 1) and Wales. Unfortunately, even though private measures to mitigate flood risk have been actively promoted, the uptake remains low (Harries, 2008; Soane et al., 2010).

In a study among flood affected households in England, Lamond, Proverbs, and Hammond (2009) found that the people that do take measures, usually take measures that are not too costly. Similarly, Joseph et al. (2015) found that the main reason for the lack of implementation of private measures is the lack of belief in the financial benefits of measures. They also found that there is a lack of ownership of the responsibility of risk mitigation, which was also one of the main factors that influence the uptake of measures found by Soane et al. (2010). In a study on the effects of flooding on housing prices, Eves (2004) found that in the long term, flood risk only influences housing prices in areas where flood insurance is not available. The presence of structural flood defences appears to have a positive influence on the housing market in areas that are at risk of flooding. Harries (2012) found that rather than being hindered by financial barriers, the uptake of measures may be influenced by emotional processes and feelings of anxiety. In a study among small business owners in England, Harries et al. (2018) found that only if the shock of the flood event is big enough this may trigger a change in behaviour. Otherwise people will use coping strategies that keep their view of their business as a safe and successful place intact, which would not be the case if they would take measures.

In a comparison of the differences in ethnic groups at risk of flooding in England and Wales, Fielding (2018) found that in all regions of England non‐white populations are disproportionately exposed to flood risks. There are differences between the regions in terms of the inequalities in exposure and awareness of the different classes. Fielding (2012) found that the working class (defined based on census data, as is commonly used to investigate health inequalities) is more likely to be at risk of flooding than the middle class in all regions except for the Midlands. The difference between classes is highest in Anglia and the North East. In Anglia the population was found to be the least aware, with no differences between classes or risk. In the Thames region and the North East the poorest people were found to be least aware of the risk.

Research reveals that in Salford in the North West of England the perceived risk of future flood events is very low even when households had experienced flooding, which could be attributed to the low frequency of flooding in one location (Alder Forest) and the construction of structural defences in the other (the Lower Irwell valley; Kazmierczak & Bichard, 2010). Kazmierczak and Bichard (2010) investigated the willingness to pay for flood protection which revealed that it was lower than the costs of flood protection and would not be enough to pay for measures. In a survey among households in Timperley in the North West, Bichard and Thurairajah (2014) found that residents did not consider the threat of flooding to be high, even after experiencing flooding. This was partly due to the structural defences that had recently been increased by the Environment Agency. Here the reasons for not undertaking measures constituted three main issues: a lack of concern about flooding and its impacts; difficulties in dealing with the information that was provided; and costs of measures.

While most research reveals that flood experience influences property level risk mitigation (Harries, 2012) and some research has been done on the factors that influence the uptake of measures in England, a lot of uncertainty remains. The surveys commissioned by Defra and the Environment Agency provide a valuable source of information. They have consistently surveyed the different Environment Agency administrative regions of England over many years, which provides us with data to compare residents' behaviour in different locations. By comparing and contrasting the behaviour of residents in the different regions we can learn from the differences and similarities and better inform flood risk management strategies. Therefore, the aim of this article is to detect if there is a difference in the relationship between flood experience and preparedness for the regions of England and to find some possible explanations as to why these differences exist.

## 2 METHODS

The analysis in this article consists of two parts. First we investigate whether there is a difference in the relationship between flood experience and preparedness for the different regions of England using Bayesian hierarchical regression. In the second part we investigate the potential influence of other factors on the flood mitigation behaviour in the regions of England.

### 2.1 Estimating the relationship between experience and preparedness

For this analysis we use data from the surveys conducted among the population at risk in the seven regions of England during the years 1997–2004. Surveys from later years are excluded, because they are not consistent in terms of sample population and questions asked. The only inconsistency between the surveys conducted from 1997 to 2000 and 2001 to 2004 is a change in the sample population from only those with flood warning provision, to also including those properties without that service. To make the data comparable across all years, correction was undertaken to account for the differences in the surveyed sample (see Appendix A). Since the surveys were (mostly) consistent and conducted among the same population we assume the data of all surveys can be treated as a pooled cross‐sectional data set. From the surveys we obtain the percentage of respondents that have experienced flooding, which we call the level of flood experience (*E*), and the percentage of respondents that have taken at least one measure to prepare for future flooding, which we call the level of preparedness (*P*) for each of the seven regions of England (see Figure 1). More information about the surveys can be found in Appendix A.

The differences in the relationship between flood experience (*E*) and preparedness (*P*) between the regions of England are investigated, by applying a Bayesian hierarchical beta regression (see Appendix B) with *E* as the explanatory variable and *P* the dependent variable. The model is described with the following equations:

(1) $$P_{i,j}\sim \text{beta}\left(\mu_{i,j}\sigma ,\left(1-\mu_{i,j}\right)\sigma \right)$$

(2) $$\mu_{i,j}=\frac{e^{{\tilde{\mu }}_{i,j}}}{1+e^{{\tilde{\mu }}_{i,j}}}$$

(3) $${\tilde{\mu }}_{i,j}=\alpha_{i}+\beta_{i}E_{i,j}$$

(4) $$\alpha_{i}=\alpha +\tau_{\alpha }{\tilde{\alpha }}_{i}$$

(5) $$\beta_{i}=\beta +\tau_{\beta }{\tilde{\beta }}_{i}$$

where *P*_*i*,*j*_ is the average level of preparedness of region *i* in year *j* and *E*_*i*,*j*_ is the average level of experience of region *i* in year *j*. The parameter *μ*_*i*,*j*_ is the mean level of preparedness in region *i* and year *j*, while *σ* is inversely related to the dispersion around the mean. We use *E*_*i*,*j*_ to calculate ${\tilde{\mu }}_{i,j}$ (Equation 3), which is then transformed to the interval between zero and one using an inverse logit to obtain *μ*_*i*,*j*_ (Equation 2). In this way, we are assuming that the regions behave differently but are related to each other. Instead of using only information from region *i* to estimate the parameters that determine its behaviour, this allows us to use the data that is available for the other regions as well. Of course this only holds under the assumption that the behaviour of residents in the regions of England is not completely unrelated. We believe that this is a reasonable assumption, since the regions are all part of the same country, with the same flood risk management structure. After estimating the values of the parameters *α* , *τ*_*α*_ , *β* , *τ*_*β*_ and the ${\tilde{\alpha }}_{i}$s and ${\tilde{\beta }}_{i}$s we can calculate the expected mean level of preparedness *P* for a given region with a given level of experience *E* .

We use uninformative normal priors for the parameters *α* (mean 0, standard deviation 1,000), *τ*_*α*_ (mean 0, standard deviation 100), *β* (mean 0, standard deviation 1,000) and *τ*_*β*_ (mean 0, standard deviation 100) and a halfnormal prior for *σ* (scale parameter 1,000). The influence of the prior distribution on the posterior distribution was tested and found to have no effect. The priors for ${\tilde{\alpha }}_{i}$ and ${\tilde{\beta }}_{i}$ are normal distributions with mean zero and standard deviation one, since they only account for the difference between regions. The size of the variation is determined by *τ*_*α*_ and *τ*_*β*_. The Bayesian inference is performed using Stan (Carpenter et al., 2017).

### 2.2 Other factors influencing private flood mitigation

After investigating the differences in the relationship in experience and preparedness we explore the presence of other factors that have been found to influence flood mitigation behaviour and whether there is a difference in influencing factors between the regions of England.

The reports on the surveys that were conducted among the at risk population that were used to estimate the relationship between experience and preparedness do not provide enough detail on other factors. However, in addition to surveys among the at risk population, the Environment Agency conducted post event surveys at different flood affected locations in England. Factors that have been found to influence flood mitigation behaviour in previous studies are matched with one or more survey questions. These surveys are focused on information received and actions taken during the event. We only consider those factors for which survey questions are available and for which survey questions were asked consistently in all surveys. These factors and the corresponding survey questions are reported in Table 1. Surveys were conducted in June 1998 in the Midlands, North East and Southern regions; in November 1998 in the Anglian, Midlands and Thames regions; in July 1999 in the Midlands, North East and North West regions; and in August 2000 in the Midlands, Southern, North East and Thames regions. Unfortunately, no surveys were conducted in the South West region. Therefore, this region is excluded from this part of the analysis. For more details about the surveys see Appendix A.

**TABLE 1:** Factors that are observed to be of influence on private flood mitigation behaviour, adapted from Bubeck, Botzen, & Aerts, 2012

Factor	Paper	Matching survey questions
Experience		
(severity of) damage suffered	Takao et al. (2004) Miceli et al. (2008) Grothmann and Reusswig (2006)	Where did your property flood? Approximately, how high above floor level did the water reach?
Experience with evacuation	Botzen et al. (2009) Botzen and van den Bergh (2012)	Advised to move yourself and others in household to a safe place. Acted on advise to move yourself and others in household to a safe place. Acted without advise to move yourself and others in household to a safe place.
Socioeconomic and geographic variables		
Household size	Kreibich et al. (2005) Zaalberg et al. (2009) Thieken et al. (2007)	Number of people in household. Number of employees.
Ethnicity	Lindell and Hwang (2008)	English is first language.
Age	Grothmann and Reusswig (2006) Botzen et al. (2009) Miceli et al. (2008) Lindell and Hwang (2008) Knocke and Kolivras (2007) Zaalberg et al. (2009)	Age group.
Gender	Grothmann and Reusswig (2006) Botzen et al. (2009) Botzen and van den Bergh (2012) Miceli et al. (2008) Lindell and Hwang (2008) Knocke and Kolivras (2007) Zaalberg et al. (2009) Duží et al. (2017)	Sex.
Hindrances for private flood mitigation		
Missing knowledge about measures	Scolobig et al. (2012) Poussin et al. (2014) Hanger et al. (2018)	I was given enough information about what to do. I did not understand what I was supposed to do. The information I was given was clear.
Coping appraisals		
Self‐efficacy	Zaalberg et al. (2009)	I felt fully prepared when the flood happened.

For each of the selected survey questions (i.e., influencing factors) we investigate whether there is a difference between regions and whether the differences between regions are correlated with the differences in preparedness.

We use a two proportions *z*‐test (see Appendix C for more details) to determine whether a region's value for a certain survey question is significantly different from the mean value of the other regions. The two proportions *z*‐test is a test of statistical significant difference between two proportions (see e.g., Fleiss, Levin, & Paik, 2013). For a certain question we have an average proportion of respondents responding ‘yes’ for one region. We consider this as our first sample. The second sample is the weighted average proportion of positive answers for all the other regions, with weights according to the number of respondents in each region. Then, using the *z*‐test, we calculate for each region the probability *p* that these two samples come from a different population. If *p* is lower than 5% we assume that the value for that region is significantly different from the average of the other regions.

We investigate what other factors may influence the difference in preparedness aside from experience. In order to compare the level of preparedness of the different regions, we consider the variation in preparedness across regions at the same level of experience and compare this with the variation in other factors across regions. The preparedness data that is available is at different levels of experience for the different regions. Therefore, we use the modelled preparedness for this comparison since the added value of the model is that we can compare the preparedness at any level of experience. To investigate the correlation between differences in influencing factors and the level of preparedness we calculate the level of preparedness at a level of experience of 50% based on the regression between flood experience and preparedness (see Section 2.1). We choose the level of 50% because it highlights the difference between the regions. We then calculate the correlation between the level of preparedness at 50% experience and the different potential influencing factors.

## 3 RESULTS

### 3.1 The relationship between experience and preparedness

**FIGURE 2:**
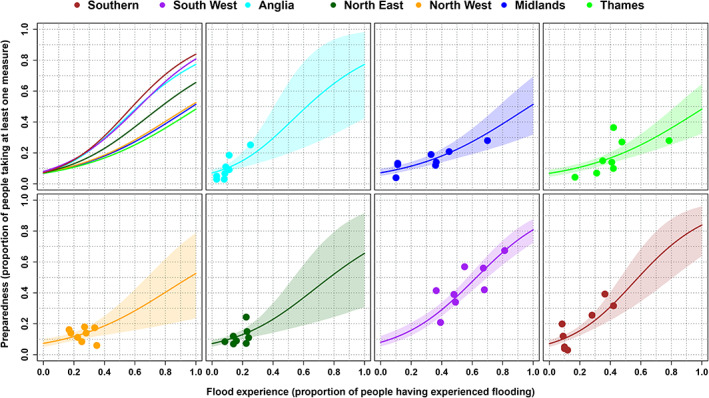
Results of the hierarchical beta regression. The top left panel shows the mean relationship between experience and preparedness for each of the seven regions. The other panels show the data (dots) and mean relationship with 90% credible interval for the different regions

Figure 2 shows the results of the hierarchical beta regression, with on the *x*‐axis the experience (*E*), that is, the proportion of respondents that report having experienced flooding and on the *y*‐axis the preparedness (*P*), that is, the proportion of respondents that report having taken at least one measure to prepare for flooding. In the top left panel we have plotted the estimated mean relationship for each of the regions. The other panels show the estimated mean relationship between experience and preparedness, the 90% credible bounds (uncertainty bounds as used in Bayesian statistics) and the data points for each of the regions separately.

The preparedness at an experience of 0 varies from 0.067 to 0.08. This indicates that in all regions a small (and similar) amount of people take measures without any prior experience of flooding. At a level of experience of 0.5, the differences are larger, with levels of preparedness of 0.41 in the Southern region, 0.39 in Anglia, 0.38 in the South West, 0.29 in the North East, 0.23 in the North West, 0.22 in the Midlands and 0.21 in the Thames region. At a level of experience of 1 the differences are largest, with levels of preparedness of 0.84 in the Southern region, 0.81 in the South West, 0.77 in Anglia, 0.66 in the North East, 0.53 in the North West, 0.51 in the Midlands and 0.48 in the Thames. At this level of experience the uncertainties are also largest, with the 90% credible bounds ranging from 0.63 to 0.96 for the Southern region, 0.72 to 0.88 for the South West, 0.41 to 0.98 for Anglia, 0.30 to 0.91 for the North East, 0.22 to 0.78 for the North West, 0.33 to 0.69 for the Midlands and 0.32 to 0.65 for the Thames region.

Looking at the estimated mean relationship between experience and preparedness (represented by the solid coloured lines), households in the regions Southern, South West and Anglia appear to increase their preparedness more when they experience flooding compared to the other regions. The North East shows an average increase in preparedness and the North West, Midlands and Thames regions have a lower increase in preparedness for a certain increase in experience. However, households in the regions Anglia, North West and North East report a low flood experience and therefore the data availability is low for higher levels of experience, resulting in wide credible bounds at higher levels of experience. If we consider these credible bounds we cannot say with certainty whether there is a difference in behaviour for the regions of Anglia, North West and North East.

### 3.2 The influence of other factors on flood mitigation behaviour in England

**FIGURE 3:**
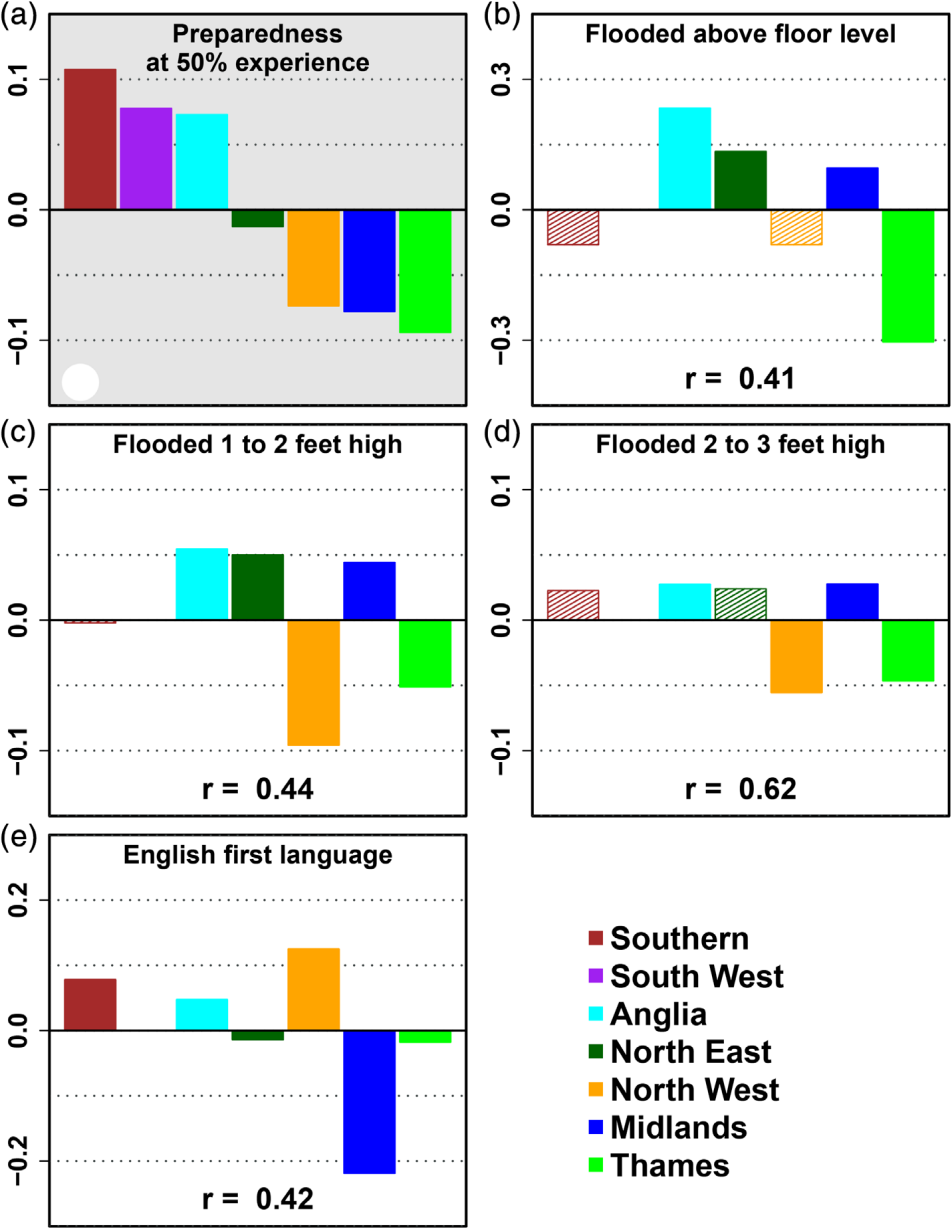
Differences in selected explanatory variables for the regions of England. Values show the difference from the mean value for England (i.e., the mean value of all regions). Panel a gives for each region the difference in the regional and mean level of preparedness for a level of experience of 50% of the population as calculated with the regression model. The other panels show for each region the difference from the mean of all regions for the selected questions. Bars with values of which the difference is significant at a level of 5% are plotted with a solid fill. Pearson's *r* values are reported at the bottom of each panel

We investigated several factors that have been previously been reported as influencing private flood mitigation behaviour. Table 2 gives the correlation of the investigated factors with the preparedness at an experience level of 50% (calculated with the model presented in Section 2.1) and the number of regions for which values are different from the mean value across regions at a significance level of 5%. Those factors with a correlation above 0.4 and at least four regions that are different from the average are plotted in Figure 3 and are indicated with grey shading in Table 2.

**TABLE 2:** Factors that may influence flood mitigation behaviour. Correlation of the factor with preparedness levels at 50% experience and the number of regions that are different from the weighted average of the other regions at a significance level of .05

Factor			Correlation (Pearson's *r*) with level of preparedness at 50% experience	Number of regions different from mean at *p* < .05
*Experience*				
Where did your property flood?		Above floor level	0.41	4
		Below floor level	−.06	5
		Garage	.41	3
		Other outbuildings	.01	4
		Garden	.34	4
		Drive	.22	3
Approximately, how high above floor level did the water reach?		Less than 1 ft	.2	2
		1–2 ft	.44	5
		2–3 ft	.62	4
		More than 3 ft	.06	4
Advised to move yourself and others in household to a safe place.			−.37	2
Acted on advise to move yourself and others in household to a safe place.			.34	5
Acted without advise to move yourself and others in household to a safe place.			−.31	4
*Socioeconomic and geographic variables*				
Number of people in household.		1	−.02	3
		2	.3	3
		3	−.41	2
		4	.11	4
		5	.16	5
Number of employees.		1–9	−.26	4
		10–24	−.26	2
		25–100	−.29	4
		101 or more	−.68	3
English is first language.			.42	6
Age group.		16–24	−.3	0
		25–34	.1	2
		35–44	.78	2
Sex.		Male	.21	2
		Female	.25	1
*Hindrances for private flood mitigation*				
I was given enough information about what to do.			.12	4
I did not understand what I was supposed to do.			−.24	1
The information I was given was clear.			−.37	3
*Coping appraisals*				
I felt fully prepared when the flood happened.			.09	4

*Note*: Factors that have a correlation above .4 and more than four regions that are different are coloured grey.

We find that only for some factors related to the severity of the flood experience (location and level of flooding) and for the factor English as a first language there is a significant difference for at least four regions and a correlation with preparedness higher than .4. The other factors do not appear to have an influence on the differences in private flood risk mitigation behaviour across the regions of England. When we look more closely at the values of these factors for the different regions (Figure 3), we clearly see that in regions where more respondents experienced flooding above floor level that went from to 1 to 3 ft the preparedness is higher. The severity of the event thus appears to be of influence on flood risk mitigation behaviour. Panel e of Figure 3 shows that in regions that tend to have less respondents whose first language is English tend to have lower preparedness.

## 4 DISCUSSION

We find that there is a positive relationship between experience and preparedness and the strength of this relationship varies for the different regions. Even though we do not have many data points for each region separately, the hierarchical regression combines information for all regions, thereby reducing the uncertainty in the regression. It could be argued that a limitation of this study is the fact that we have used data from surveys that were conducted in the period between 1997 and 2004, because this is already a while ago. However, it reflects relationships that may well persist for residents that have not received additional mitigation since this period. Research in England has shown that on average the uptake of private measures does not change significantly despite current interventions (Harries, 2008; Harries, 2012; Thurston et al., 2008). In a survey that was conducted among the British public in 2014, Capstick et al. (2015) found that only 1% of the respondents had bought flood protection products, 1% had sought advise on how to protect their property against flooding, 2% had prepared a plan of what to do in case of a flood, 4% had signed up for flood warnings and 26% had made sure they had insurance cover for flooding. Another limitation that follows from the restricted nature of the data that was available for this study is that we do not account for the differences in types of precautionary measures. Some measures are more expensive and more difficult to implement than others and therefore reasons for not implementing measures may be different depending on the measures. Despite the limitations, these surveys do provide a consistent data set in space and time, from which we can learn something about the average differences in public behaviour between regions. An analysis of more recently collected data could investigate whether the differences between regions remain the same and whether the type of measures that are implemented changes over time.

The importance of the severity of an experienced flood event (e.g., whether properties got flooded above floor level or experienced higher inundation depths) for triggering changes in preparedness is in line with findings by Harries et al. (2018) and Thieken et al. (2007). Contrary to Scolobig et al. (2012) and Duží et al. (2017) we find that the provision of information does not affect the uptake of private measures. However, this is similar to findings in other studies that were undertaken in England that find that the provision of information alone is not enough (Soane et al., 2010) or that people may have difficulties dealing with the amount and variety of information they receive (Bichard & Thurairajah, 2014).

The analysis of the additional factors that may influence the uptake of measures is an exploratory analysis, even though it is based on factors that have been found to influence the uptake of measures in previous work (Bubeck, Botzen, Kreibich, et al., 2012; Bubeck, Botzen, & Aerts, 2012). In the analysis we use six points (i.e., the six values for each of the regions) to find correlations between preparedness at 50% experience and the influencing factor. This does not give a very reliable estimate of the correlations and therefore has high uncertainty. In addition, due to the nature of the available data it is not possible to include these factors in the regression model. This is a limitation, since these factors do not simply influence the relationship between experience and preparedness, but rather preparedness is a consequence of a complex interplay between all factors. However, the analysis of the influencing factors is an attempt to find an indication of other factors that influence the uptake of precautionary measures which may provide an explanation as to why the relationship between experience and preparedness is different in the different regions. In future research these hypotheses can be tested using other (and more) data and other methods, like for example, regression analyses or socio‐hydrological models.

## 5 CONCLUSION

The analysis in this article reveals that the increase in preparedness with increasing flood experience is low in the Midlands, North West and Thames regions. In the North East the increase in preparedness is moderate and residents in the Southern and South West regions of England have a higher tendency to increase their preparedness with an increase in flood experience. An investigation of additional factors that may influence the uptake of private measures shows that the only factors that may be able to account for the differences in regions are the severity of the experienced flooding and whether English is the first language or not. Other factors that have been previously found to have an influence do not appear to be significantly different for the regions of England. These hypotheses can be tested further in future research to gain more insight into the causes of different behaviour between the different regions. By contrasting and comparing case studies using data that has been collected in a consistent way, we can learn from the differences and similarities and better inform flood risk management strategies.

## Supporting information


Appendix

## Data Availability

The authors would like to thank the Environment Agency for the provision of the survey data that was used for the analyses in this paper. The data that support the findings of this study may be requested from the Environment Agency.
