# Supplementary material for: A comparative analysis of the relationship between flood experience and private flood mitigation behaviour in the regions of England
Source: J Flood Risk Manag. 2021 Feb 6;14(2):e12700. doi: 10.1111/jfr3.12700 (PMC8641382; doi:10.1111/jfr3.12700)
Supplement: Supplementary file 1 — Appendix [file JFR3-14-e12700-s001.docx]

**Appendix A**

Since 1997, the Environment Agency has monitored people’s knowledge about flood warnings, the environment agency, awareness and people’s preparedness to deal with floods (BMRB, 2001^[[1]](#footnote-1)^). BMRB has conducted surveys among property owners in the seven regions of England and Wales. The research has consisted of three different parts:

- Surveys among the entire population of England and Wales to monitor the awareness of the Environment Agency’s responsibility for managing flood risk, issue flood warnings and of the flood warning system.
- Surveys among the population of England and Wales that is at risk of flooding. These surveys investigate the population’s awareness of their risk, of the warning system, but also their preparedness to deal with flooding.
- Post event surveys were conducted among the population that had suffered from a flood event.

In this study we use the surveys that were conducted among the at risk population in the years 1997 to 2004 to study the relationship between flood experience and preparedness. After the year 2004, the Environment Agency has changed the frequency and set up of the surveys. Therefore, the later surveys are not used to determine the relationship between experience and preparedness. The survey that was closest in time and for which the complete data set was available is the At Risk survey of 2010. This survey was used to determine which variables may provide an explanation for the differences in the relationship between experience and preparedness, along with the post event surveys that were conducted in the years 1998 to 2004. The following paragraphs give some more details about the individual surveys.

*At risk surveys 1997-2004*

Table A1 gives the sample sizes for the eight surveys. The only inconsistency between the surveys conducted from 1997-2000 and 2001-2004 is a change in the sample population from only those serviced by flood warnings, to also including those properties that are at risk but not serviced. To make it comparable across all years, the data were corrected to account for the differences in the surveyed sample by multiplying it with the average percentage of people that had ever received a flood warning in 2000 divided by the average percentage of people that had ever received a flood warning in 2001. In addition, in 2002 to 2004, the reported values for the number of people that had taken at least one measures are only for those people who could name some measures when asked. Therefore, for these years, the values for the preparedness were multiplied with the percentage of people that could at least name one measure in the previous question.

**Table A1.** Sample sizes for the different regions for the surveys in the years 1997 to 2004

|  | Region sample size | | | | | | |
| --- | --- | --- | --- | --- | --- | --- | --- |
| Survey | Anglia | Midlands | North West | North East | Southern | South West | Thames |
| 1997 | 109 | 108 | 83 | 127 | 196 | 87 | 124 |
| 1998 | 159 | 83 | 114 | 157 | 300 | 69 | 142 |
| 1999 | 174 | 79 | 117 | 100 | 308 | 71 | 138 |
| 2000 | 194 | 53 | 101 | 98 | 325 | 73 | 132 |
| 2001 | 140 | 104 | 112 | 157 | 119 | 119 | 181 |
| 2002 | 179 | 99 | 100 | 134 | 117 | 93 | 186 |
| 2003 | 148 | 117 | 114 | 127 | 118 | 101 | 115 |
| 2004 | 154 | 135 | 105 | 115 | 125 | 101 | 109 |

*Post event surveys*

The questions of the Post Event surveys conducted in the years 1998 to 2000 were investigated as possible variables that influence the relationship between experience and preparedness. Sample sizes are reported in Table A2. Post Event surveys from later years were excluded, either because data was not available or because the questions were not consistent with those in the earlier years.

**Table A2.** Sample sizes for the different regions for the surveys in the years 1997 to 2004

|  | Region sample size | | | | | | |
| --- | --- | --- | --- | --- | --- | --- | --- |
| Survey | Anglia | Midlands | North West | North East | Southern | South West | Thames |
| June 1998 | - | 172 | - | 10 | 6 | - | - |
| November 1998 | 193 | 98 | - | - | - | - | 12 |
| July 1999 | - | 100 | 8 | 41 | - | - | - |
| August 2000 | - | 87 | - | 100 | 122 |  | 100 |
| Total | 193 | 457 | 8 | 151 | 128 | - | 112 |

**Appendix B**

We use Bayesian inference (Gelman et al., 2014^[[2]](#footnote-2)^) to estimate the parameters of the hierarchical model. Bayes' theorem (equation B1) can be used to derive the posterior distribution of a set of parameters from a combination of data and prior knowledge about the parameter distribution.

$$\begin{aligned} p\left( \theta|y \right)=\frac{p\left( y|\theta\right)p\left( \theta\right)}{\int p\left( y|\theta\right)p\left( \theta\right)d\theta}\#B1 \end{aligned}$$

p(θ|y) is the posterior estimate of the distribution of the parameters given the prior knowledge and the data y. p(y|θ) is the likelihood of the observed data, given the values of the parameters. This incorporates the information about the data y and the uncertainty of the data. The prior knowledge about the parameter values is incorporated in the distribution p(θ).

The integral in the denominator of Bayes' rule is in this case not analytically solvable, therefore, the posterior distribution of the parameters is approximated using a Markov Chain Monte Carlo (MCMC) simulation method. These methods sample from a probability distribution by constructing a Markov chain that has the wanted distribution (e.g. the posterior estimate of the parameter distributions) as its equilibrium distribution (see, e.g., Gelman et al., 2014^2^; Robert & Casella, 2004^[[3]](#footnote-3)^). These chains are run for a large number of steps and the states of these chains can be used as a sample from the posterior distribution. We use the software Stan (Carpenter et al., 2017^[[4]](#footnote-4)^) to perform the MCMC inference.

**Appendix C**

We use a two proportions z-test to determine whether a region’s value for a certain variable is significantly different from the mean value of the other regions. Fleiss et al. (2013, page 60^[[5]](#footnote-5)^) describe the confidence interval for the difference between two proportions. We use an adapted version to determine whether the values for the regions are significantly different from the other regions. We calculate the test statistic as follows:

$$\begin{aligned} p_{i}=\frac{s_{i}}{n_{i}}\#C1 \end{aligned}$$

$$\begin{aligned} p_{j}=\frac{s_{j}}{n_{j}}\#C2 \end{aligned}$$

$$\begin{aligned} p_{J}=\sum_{j\neq i} \frac{1}{6}p_{j}\#C3 \end{aligned}$$

$$\begin{aligned} {SE}_{i}=\sqrt{\frac{p_{i}\left( 1-p_{i} \right)}{n_{i}}+\sum_{j\neq i} \left( \frac{1}{6} \right)^{2}\frac{p_{j}\left( 1-p_{j} \right)}{n_{j}}}\#C4 \end{aligned}$$

$$\begin{aligned} z_{i}=\frac{p_{i}-p_{J}}{{SE}_{i}}\#C5 \end{aligned}$$

Where $p_{i}$ is the proportion for region *i*, $p_{j}$ is the proportion for region *j*, and $p_{J}$ is the averaged proportion for the regions *j* that are not region *i*. $s_{i}$ and $s_{j}$ are the number of positive responses and $n_{i}$ and $n_{j}$ the sample size. ${SE}_{i}$ is the standard error. The z-statistic can then be calculated as given in equation C5.

1. BMRB. (2001) Flood Warning Dissemination – Post Event Survey report August 2001 [↑](#footnote-ref-1)
2. Gelman, A., Carlin, J. B., Stern, H. S., Dunson, D. B., Vehtari, A., & Rubin, D. B. (2014). Bayesian data analysis (Vol. 2). Boca Raton, FL: CRC Press. [↑](#footnote-ref-2)
3. Robert, C. P., & Casella, G. (2004). Monte Carlo statistical methods. New York: Springer. [↑](#footnote-ref-3)
4. Carpenter, B., Gelman, A., Hoffman, M., Lee, D., Goodrich, B., Betancourt, M., et al. (2017). Stan: A probabilistic programming language. Journal of Statistical Software, Articles, 76(1), 1–32. https://doi.org/10.18637/jss.v076.i01 [↑](#footnote-ref-4)
5. Fleiss, J. L., Levin, B., & Paik, M. C. (2013). Statistical methods for rates and proportions. John Wiley & Sons. [↑](#footnote-ref-5)
